# Road Traffic Gesture Autonomous Integrity Monitoring Using Fuzzy Logic

**DOI:** 10.3390/s25010152

**Published:** 2024-12-30

**Authors:** Kwame Owusu Ampadu, Michael Huebner

**Affiliations:** Computer Engineering, Brandenburg University of Technology, Cottbus-Senftenberg, 03046 Cottbus, Germany; michael.huebner@b-tu.de

**Keywords:** Advanced Driver Assistance System (ADAS), autonomous vehicles, extended blind-spot detection, fuzzy logic, gesture recognition, in-vehicle traffic light

## Abstract

Occasionally, four cars arrive at the four legs of an unsignalized intersection at the same time or almost at the same time. If each lane has a stop sign, all four cars are required to stop. In such instances, gestures are used to communicate approval for one vehicle to leave. Nevertheless, the autonomous vehicle lacks the ability to participate in gestural exchanges. A sophisticated in-vehicle traffic light system has therefore been developed to monitor and facilitate communication among autonomous vehicles and classic car drivers. The fuzzy logic-based system was implemented and evaluated on a self-organizing network comprising eight ESP32 microcontrollers, all operating under the same program. A single GPS sensor connects to each microcontroller that also manages three light-emitting diodes. The ESPNow broadcast feature is used. The system requires no internet service and no large-scale or long-term storage, such as the driving cloud platform, making it backward-compatible with classical vehicles. Simulations were conducted based on the order and arrival direction of vehicles at three junctions. Results have shown that autonomous vehicles at four-legged intersections can now communicate with human drivers at a much lower cost with precise position classification and lane dispersion under 30 s.

## 1. Introduction

Traffic engineering addresses the functional aspects of transportation infrastructure by developing increasingly intelligent systems to ensure the safe and efficient movement of people and goods by road, rail, water, or air. Road traffic research can be credited with the development of road surface markings, traffic signs, traffic lights, and various rules and regulations that are used to calm traffic in residential and restricted areas. The successful development of intelligent systems has been achieved mainly through collaboration with electrical and computer engineering counterparts. A conventional traffic light configuration consists of a detector loop, a lighting/display device, local controllers, a master controller, communication lines, and a traffic control center, made possible by the convergence of traffic engineering, electrical engineering, and computer engineering disciplines.

Similar collaborations have led to the evolution of various driver assistance systems on the automobile. Intelligent vehicles are becoming more common as cognitive car research gains momentum in transferring mental abilities into technological systems [1,2]. Hardware can now learn through software just like humans, giving birth to various context-aware Advanced Driver Assistance Systems (ADASs) on the automobile. The car of the future is autonomous [3,4]. Drunkenness, distraction, inattentiveness, carelessness, tiredness, and fatigue lead to handling errors that do not plague the autonomous vehicle.

Improving road traffic safety and eliminating automobile handling errors requires a concerted effort from Original Equipment Manufacturers (OEMs), industrial practitioners, and researchers, as well as policy makers. The annual budget at state and local levels in the United States of America (U.S.A) for operating and maintaining signalized intersections is in excess of $1.23 billion, as indicated by the Traffic Signal Benchmarking and State of the Practice Report, 2019. To this end, self-correcting road traffic technologies and policies are being progressively designed and refined, leading to Artificial Intelligence (AI) applications for monitoring road traffic situations that have made auto-piloting and navigation more reliable [5,6].

Road intersections serve a useful purpose in changing the direction of travel by guiding vehicles from one lane into the other. However, they present opportunities for conflicts [7,8]. All manners of road users would want to cross the intersection first and in the shortest possible time. This leads to a situation where all road crossing participants want to be at the same place at the same time. The controller of an approaching vehicle must therefore be apt and very alert in entering and leaving an intersection, as a digression of thought can lead to catastrophic and unintended consequences. Conventionally, there have been three forms of conflict control at intersections.

Passive control implements basic rules to ensure the free flow of low-volume traffic from one lane into the next. The dispersion of low-volume traffic is mainly dependent on road traffic regulations, traffic signs, and road markings. In semi or partial control, physical barriers are used to channel traffic flow from conflicting points into milder areas. These physical channels may include islands or traffic rotaries. Active conflict control involves the imposition of time and direction constraints on arriving and departing vehicles in heavy-traffic environments. Here, traffic control signals may be used to impose time constraints whereas grade- or level-separated roads may be used to activate direction constraints. The latter has an added advantage of high-speed crossing, especially on highways.

Gesture detection and recognition on a traffic scene presents a way for humans to interact with the automobile using body language. The possibility is attained through computer vision by the use of processing systems running neural networks or rule-based algorithms. In the automotive environment, a gesture is used to either manipulate a system or convey a message. These upper body movements usually originate from the head and/or hand(s). A gesture is called static if the processing of a single image is sufficient for its interpretation. This is loosely known as a posture. If such a gesture is cast over a timespan, the message will be same at the beginning and end of its propagation. Dynamic gestures, on the other hand, involve the processing of a sequence of postures. A gesture that is acknowledged to trigger an event or carry out a command is recognized as an offline gesture.

Traffic scene-understanding technology uses sensory data collection, analyses, and interpretation. The cost in hardware, software, and energy demands towards correctly identifying gestures under all weather, though not fully accomplished, is increasing. A number of approaches used to detect and recognize gestures are presented in [9,10,11,12,13]. Traditionally, Light Detection and Ranging (LiDAR), Radio Detection and Ranging (Radar), Ultrasonic, and Vision (camera) systems with supplemental sensors have been employed for the mitigation of traffic scene contingencies [14,15,16]. In general, inertial measuring units may help cars to detect their physical movements and stabilize themselves for safety purposes while Global Positioning System (GPS) units help in obtaining place and time information on local maps. Imaging sensors have been used to identify road obstacles, read road signs, and recognize other moving vehicles and pedestrians. Lidar sensors have been more accurate in determining vehicle positions, whereas radar sensors have been good at estimating speeds of moving objects. By fusing data from GPS, lidar, radar, video cameras, and other sources, efficient algorithms have been implemented to detect poses of drivers, riders, police officers and traffic marshals under varying climatic conditions [17,18,19] in answer to some road safety recommendations.

Junctions without traffic lights outnumber those with traffic lights [20]. When yielding the right of way at crossroads without traffic lights, exaggerated hand gestures are used by human drivers. The autonomous car by nature is devoid of this ability even when fully occupied and is more disadvantaged under bad weather when on-board cameras and sensors cannot capture an approaching driver’s hand signals accurately. The driverless-car-to-human-driver gesture communication gap in gridlock situations at intersections needs addressing. Bad weather and its associated power failure compounds the problem of intersection communication between classic vehicles and autonomous vehicles that rely on camera feed, even in the presence of roadside traffic lights. Prevailing sensor and algorithmic limitations also make gridlock conflict resolution at crossroads a difficult task among manned and unmanned vehicles.

Fuzzy logic is widely used in the automobile industry, having the advantage of using simple rules for the optimization of control systems that incorporate engineering expertise. It is also code-space-efficient and can be used for a Gesture Autonomous Integrity Monitoring (GAIM) in-vehicle traffic light solution to gridlock situations that bypasses most of the sensor and heavy algorithmic limitations in the literature. This could be accomplished by creating a vehicular network with microcontrollers that run the same program and communicate traffic priorities wirelessly at road intersections. Such a traffic control technology, requiring no large-scale or long-term storage such as the driving cloud platform and being decoupled from roadside infrastructure, will be cheap to acquire, easy to install, and shareable among autonomous and regular vehicles. The authors of [21,22] concluded that an in-vehicle traffic light technology could properly manage traffic flow at unsignalized intersections.

The major contribution of this research is the design and implementation of an in-vehicle traffic light system based on fuzzy logic that does not rely on roadside units to resolve grid lock conflicts at unsignalized four-legged intersections involving autonomous and classical vehicles. The system uses GPS coordinates in a self-organizing vehicular network to classify vehicle positions at the intersections and vote to disperse cars in one lane. To the best of our knowledge, this is the first in-vehicle traffic light system underpinned by fuzzy logic that operates independently of any roadside infrastructure.

## 2. Review of Related Work

The traffic safety improvement strategy in [23] required a combination of roadside controller nodes, short-range devices forming vehicular networks, and intersection-based traffic lights to adapt to changing timing schemes. In this system, the controller nodes set the traffic light phases while the vehicles respond to the changing lights. The introduction of in-vehicle communicating devices allows for the exchange of information through a mobile ad hoc network in place of lane-embedded loop detectors to adapt to its dynamic timing, which comes with significant cost savings. Approaching vehicles exchange information with traffic lights which, in turn, communicate with adjacent traffic lights at other intersections. The most important metrics obtained from these exchanges include control delay and queue length, which are used to generate processes such as phase skipping, extension, or interruption. For want of a better simulator, a discrete Vehicular ad hoc Network (VANET) simulator was developed by integrating vehicular mobility and wireless transmission. The measure of efficiency was performed on existing pre-timed traffic control signals and compared to the adaptive strategy combining the controller nodes, approaching vehicles, and the traffic lights. The adaptive system outperformed the pre-timed system and recorded faster congestion recovery at intersections. Vehicular mobile ad hoc networks can therefore be used in conflict resolution at unsignalized intersections involving autonomous vehicles.

Speed adaptation characteristics in autonomous dynamic driving were utilized in [24] to guarantee safe intersection crossing involving a human-driven vehicle and a driverless vehicle. Twenty-four fuzzy rules with four inputs were used to determine the velocity of the autonomous vehicle with respect to that of the driven one. In addition, three inputs were used to establish the exact and relative positions of both vehicles. Though their means of data exchange is not specified, the system is said to receive horizontal and vertical coordinates of both vehicles for analysis carried out in a Takagi–Sugeno–Kang fuzzy inference engine, the implementation of which is also not described outside of the MATLAB environment. To support the lane crossing maneuver at the collision point, two path-tracking models were used to enhance the output speed. Namely, a Proportional Integral Derivative (PID) and a Model Predictive Control (MPC). Based on the Root Mean Squared Error (RMSE) values from both controllers, the MPC was said to improve the intersection crossing maneuver better than the PID controller. Simulation results confirm the success of this approach in varying the speed of the autonomous vehicle appropriately to avoid collision and near-collision incidents. Such a system will be difficult to replicate and implement for conflict resolution in larger, mixed-traffic scenarios.

The idea of a Virtual Traffic Light (VTL) was first proposed in [20] as a way to transform roadside traffic lights into an in-vehicle system. It required all vehicles to be furnished with Dedicated Short-Range Communication (DSRC) devices, a digital road map, and a GPS input. Making use of a database to determine the appropriate intersections for VTL activation. The strategy depended on an elected leader among approaching vehicles to play the role of a short-term traffic controller. The stopping distance of the leading vehicle was not established and was assumed to be as close to the intersection as needed. The failure of the leading central controller could subsequently defeat the entire purpose of the self-organized in-vehicle traffic control system, and therefore, a different approach such as the Gesture Autonomous Integrity Monitoring (GAIM) technology is needed.

The feasibility of a DSRC-based Virtual Traffic Light (VTL) system was explored in [25]. The use of an On-Board Unit (OBU) was preferred over Road Side Units (RSUs) for its data broadcast. However, the leader-selection approach in [20], detrimental to a truly ubiquitous self-organizing traffic control system, was repeated here. Large memory requirements would also be necessary for such heavyweight algorithms. The implementation choice involved the use of a Basic Safety Message (BSM) in the sensing step, WAVE Short Message (WSM) in the leader selection step, and Signal Phase and Timing (SPaT) for broadcast. The WSM was also used during the handover to newly elected leaders. Release of the elected leader was consequential to broadcast stoppage. A tablet was also added for the visual display of the red, yellow, and green lights. The results reported included the system viability in Non-Line of Sight (NLoS) situations for real world applications.

The VTL approach in [21] was also based on [20], the difference being the projection of traffic information on the windshield. The introduction of additional images to its custom graphical user interface to prompt users approaching intersections of traffic situations from distances of up to 200 m had an additional advantage over previous VTL implementations. First, a traffic-signal-ahead indication was given, followed by signs indicating priorities at the intersection. Tests were also performed to compare the impact of VTL and roadside mounted traffic lights on the deceleration of vehicles approaching the intersection. Higher deceleration values were recorded in the case of the VTL over that of the conventional traffic lights. As with all new technologies, further testing would be necessary to improve user acceptance comparable to that of the existing traffic control devices.

Unfortunately, none of the foregoing approaches to in-vehicle traffic control or the VTL took weather elements into consideration. Research efforts have successfully addressed challenges involving space segment radiation, satellite clock to receiver clock matching, ionospheric and tropospheric interference, multipath interference, and some handling errors to the extent that the GPS receiver can perform geolocation under all climatic conditions. Accurate global positioning systems guarantee that a vehicle’s location at all road intersections can be determined. Receiver equipment are getting smaller and cheaper. Some are so accurate that their locations can be pinpointed within 1 to 5 cm accuracy [26]. A truly ubiquitous in-vehicle traffic light system based on fuzzy logic can rely on existing GPS capabilities for accurate input data under all climatic conditions.

## 3. Foundations for System Design

### 3.1. Fuzzy Logic Foundations

In classical (Boolean) logic, either a statement is true, or its negation is false. That is to say, an autonomous vehicle is either at an intersection or it is not. However, in reality, the vehicle may be at the intersection, close to the intersection, or far away from it. Fuzzy logic accommodates partial inclusion beyond the binary extremities of yes and no or true and false. It incorporates such intermediate possibilities between yes and no surrounding a vehicle at an intersection as being at the intersection, close to the intersection, or far away from the intersection. These intermediate possibilities are referred to as the degree of belonging to a yes (1) or a no (0) in the closed interval [0,1]. The truth of a statement in fuzzy logic depends on this degree.

Data from sensors form crisp input and are translated by a fuzzification interface also known as a fuzzifier into appropriate linguistic values using membership functions stored in a knowledge base. A fuzzy inference engine maps the linguistic values to corresponding fuzzy outputs stored in a knowledge base. The knowledge base, derived from an expert’s experiences, is a collection of “if—then” rules and a database containing membership functions and conjunction/disjunction elements used in forming the fuzzy rules. A defuzzification interface then translates the results from the inference engine back into the form in which the input from sensors were obtained: crisp values.

There are two main fuzzy inference methods: Mamdani and Sugeno. While the Mamdani approach to fuzzy inference results in a fuzzy set which requires a defuzzification step, the Sugeno inference outputs a mathematical expression of the result or a weighted average of the rule strengths, and hence, no defuzzification step is required. The Mamdani-type fuzzy inference system was chosen for this work.

### 3.2. Mathematical Foundation

Given that there are n identical cars in a gridlock at a four-legged intersection, n≥4, where n is a natural number. It can be shown through mathematical induction that cars on one lane can be dispersed through a voting scheme. When n=4, there will be one car on each lane. Let $P\left(m\right)$ be the number of cars on the lane, where m is a natural number. $P\left(m=1\right)$ holds true for the Leading car, since three cars can vote to disperse one car. When n≻4, there can be more than one car on the lane. Let k be the arbitrary index of a car on the lane. Assuming that $P\left(m=k\right)$ is true, want to show that $P\left(m=k+1\right)$ is also true for the next car on the lane. If $P\left(m=k\right)$ is true then the car will vacate its position for the next car after receiving the green light vote. That is $P\left(m=k+1\right)$ will be voted for to leave the intersection. Hence $P\left(m=k+1\right)$ is also true. Because $P\left(m=1\right)$ is true for n=4 and $P\left(m=k\right)\Rightarrow P\left(m=k+1\right)$ is true for n≻4, $P\left(n\right)$ must be true for all n≥4. Therefore, the number of vehicle nodes per intersecting lane required for the fuzzy classification model inferred from the principle of mathematical induction should be n≥2. Thus, at least two microcontrollers per lane will suffice for the ad hoc vehicular network, each vehicle hosting one microcontroller, resulting in at least eight microcontrollers for addressing the gridlock conflict.

**Figure 1:**
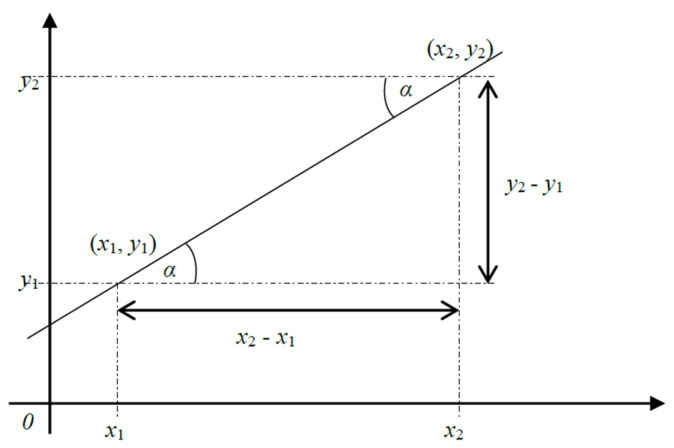
Car positions on the Cartesian plane.

Four input variables can be determined from the mutual characteristics of the positions of two cars $\left(x_{1},y_{1}\right)$ and $\left(x_{2},y_{2}\right)$ with corresponding membership functions. The positions of two cars $\left(x_{1},y_{1}\right)$ and $\left(x_{2},y_{2}\right)$ can be displayed graphically on the cartesian coordinate system as follows, Figure 1.

The straight line passing through the two points in Figure 1 has a gradient m, (1), that remains constant along its entire length. By definition,

(1) $$m=\frac{y_{2}-y_{1}}{x_{2}-x_{1}}$$

In a ratio, $y_{2}-y_{1}$ is referred to as the numerator while $x_{2}-x_{1}$ serves as the denominator. The shortest distance between the two points will be given by the length l of the line, (2), connecting them at an angle of inclination α to the horizontal.

By definition,

(2) $$l={\left({\left(x_{2}-x_{1}\right)}^{2}+{\left(y_{2}-y_{1}\right)}^{2}\right)}^{\frac{1}{2}}$$

Thus, the mutual characteristics shared by the two points are length (distance), numerator, denominator, and an angle of inclination. These characteristics, namely, distance, numerator, denominator, and angle of inclination, are necessary and sufficient for their mutual description and will serve as input to the fuzzy logic model. However, the car’s GPS coordinates are based on the geodetic coordinate system. For vehicles whose wheelbase remain on the ground, the altitude component can be ignored (set to one unit). Hence, GPS location coordinates would be transformed into Cartesian coordinates, using (3) and (4).

(3) x=radOfEarth*cos(latitudeGPS)*cos(longitudeGPS)

(4) y=radOfEarth*cos(latitudeGPS)*sin(longitudeGPS)

where radOfEarth is the radius of the Earth, latitudeGPS and longitudeGPS are the real-time latitude and longitude values obtained from the GPS module connected to a microcontroller respectively.

Given a four-legged intersection, four linguistic output variables will suffice to indicate vehicle positions on its northern, eastern, southern and western lanes. It is necessary to establish the relative position of each car from the next. Thus, one car can be *behind* the other, one on the *left*, one on the *right* or one *ahead* for n≥4, where n, a natural number is the number of cars in gridlock conflict at the intersection. Four output variables (East, North, West and South) each with four membership functions (Ahead, Left, Right and Back) will be needed.

Similarly, a car can be at the intersection, close to the intersection or far from it, resulting in three membership functions for the distance variable. The value of the denominator may tend towards −∞, 0, or +∞ just like the numerator. Thus, the denominator and the numerator will each have three membership functions. To cater for single entry and single exit lanes at the intersection, each quadrant on the Cartesian coordinate system will be divided into two, resulting in eight membership functions for the angle of inclination variable.

Let the four inputs to the fuzzy inference system be:

$$x_{1}=A_{1i},\;i=1,2,3$$

$$x_{2}=A_{2j},\;j=1,2,3$$

$$x_{3}=A_{3k},\;k=1,2,3$$

$$x_{4}=A_{4l},\;l=1,2,3,\ldots ,8$$

Let the four output positions on the four-legged intersection be represented by:

$$y_{1}=A_{1u},\;u=1,2,3,4$$

$$y_{2}=A_{2v},\;v=1,2,3,4$$

$$y_{3}=A_{3w},\;w=1,2,3,4$$

$$y_{4}=A_{4z},\;z=1,2,3,4$$

Then fuzzy relationship

$$R=\cup \left(A_{1jkl}\times A_{2ikl}\times A_{3ijl}\times A_{4ijk}\right)\times \cup \left(A_{1vwz}\times A_{2uwz}\times A_{3uvz}\times A_{4uvw}\right)$$

After defuzzification the output

$$O=\frac{\sum_{i=1}^{3}\sum_{j=1}^{3}\sum_{k=1}^{3}\sum_{l=1}^{8}d_{ijkl}\mu_{A1i}\left(x_{i}\right)\mu_{A2j}\left(x_{j}\right)\mu_{A3k}\left(x_{k}\right)\mu_{A4l}\left(x_{l}\right)}{\sum_{u=1}^{4}\sum_{v=1}^{4}\sum_{w=1}^{4}\sum_{z=1}^{4}\mu_{A1u}\left(x_{u}\right)\mu_{A2v}\left(x_{v}\right)\mu_{A3w}\left(x_{w}\right)\mu_{A4z}\left(x_{z}\right)}$$

where: $A=\{(x,\mu_{A}(x)):x\in X\}$. X is the universe of discourse, $\mu_{A}(x)$ is called the membership value, the grade of membership or the degree of belonging of x∈A and $\mu_{A}(x)$ lies in the closed interval [0,1].

## 4. System Design Methodology

**Figure 2:**
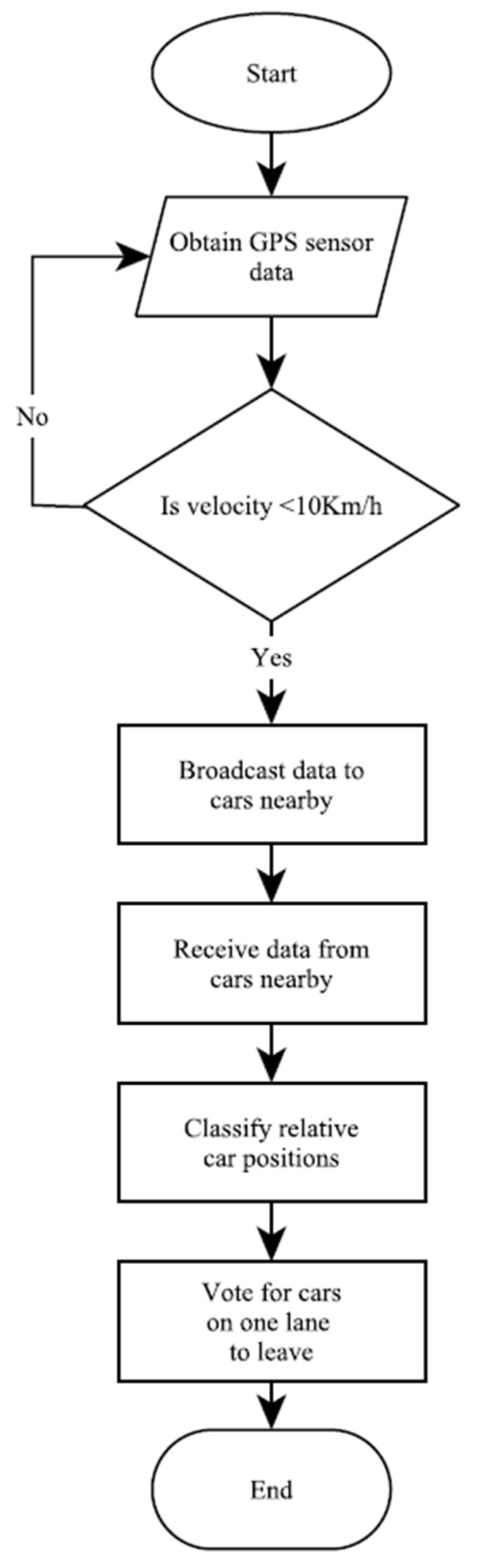
Priority assignment sequence.

The Road Traffic Gesture Autonomous Integrity Monitoring system is an in-vehicle traffic light system. It obtains on-board GPS sensor data (Figure 2) and broadcasts them to nearby cars in a self-organizing vehicular network at a four-legged intersection. The vehicular network is built on ESP32 microcontrollers and uses the ESPNow broadcast feature to share data. To enhance location classification, each microcontroller is assigned the registration number of the vehicle hosting it. The data received are fed to a fuzzy inference system that determines the relative position of each decelerating or stopped vehicle at the intersection. Three of the cars then vote for one vehicle to leave without the use of gestures. All the microcontrollers operate under the same program.

Each microcontroller has an embedded unique Media Access Control (MAC) address and is able to broadcast its assigned vehicle registration number, its location coordinates, and the MAC address itself within a 250 m radius. Such data are then used to determine the relative positions at the intersection of all vehicles converging on the northern, western, southern, and eastern lanes.

In a manner similar to how human drivers in a gridlock at an intersection use gestures to indicate to cars on one lane to proceed, a voting scheme will be employed to manage the flow of traffic in that lane. The dispersion is based on control signals that are sent to three pins, namely, the *standby* pin, the *brake* pin, and the *throttle* pin. Since a car cannot accelerate and brake simultaneously, only a single pin is activated at a time. In other words, when the *digitalwrite* signal is high on the brake pin, *digitalwrite* goes low on the throttle pin. The placement of a traffic control device should ensure adequate visibility in the road user’s view. Provisions made for a human driver on the dashboard include a *red* light for movement prohibition, a *yellow* light for caution, and a *green* light for permission to move. This guarantees that the human driver notices a red light when the *brake* pin is activated and a green light when the *throttle* pin is activated, with both signals operating intermittently. Meanwhile, the autonomous vehicle can either come to a stop when the *digitalwrite* signal on the *brake* pin is high or proceed smoothly through a junction when the *digitalwrite* signal on the *throttle* pin is high. Light-Emitting Diodes (LEDs) are cheap, energy efficient, and long-lasting and can easily be incorporated into the instrument cluster panel of a road vehicle, bringing the traffic light closer to the driver. The lights can also be accompanied by audible messages and vibration of the driver’s seat to draw attention to traffic flow, an advantage which the current infrastructure-based traffic control devices cannot provide the driving and riding public.

**Figure 3:**
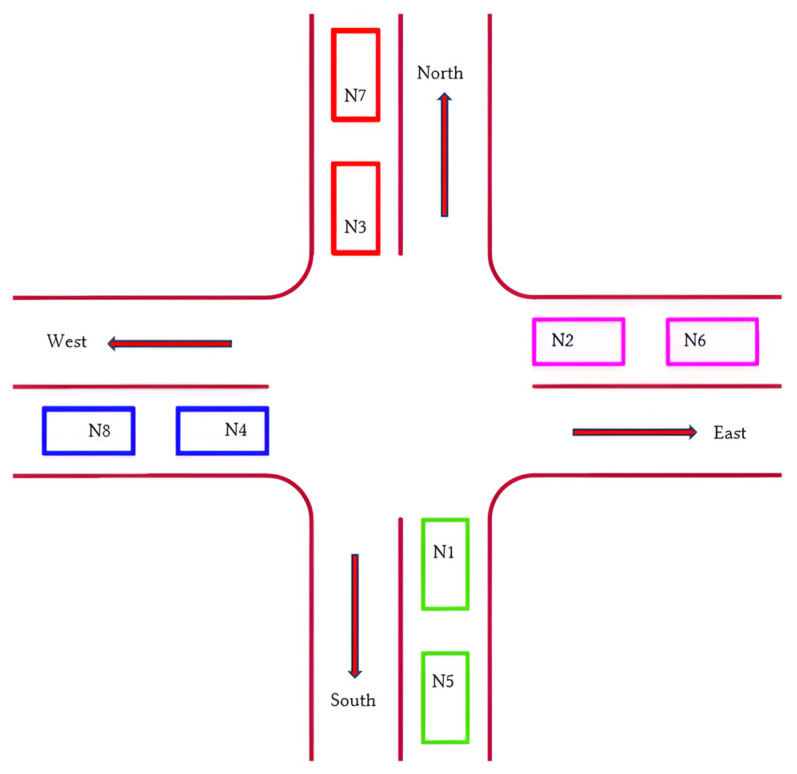
Layout of an intersection indicating north, south, west, and east lanes with two car nodes each.

A road lane of 4 m width having an orthogonal fillet radius of 2 m was used with an average car length of 4 m. The width of each car, represented by a rectangle in Figure 3, was also set at 2 m. The AutoCAD2023 software used for the design layout supports the use of real-world units.

Mutual characteristics between pairs of node location coordinates were used to establish 32 fuzzy rules for the relative position classification at the intersection. Since a trailing node could be shifted slightly to the left or right behind a leading node, Back1 and Back2 membership functions were introduced to account for such discrepancies. Four output variables were defined for the fuzzy model, each having four membership functions. Four input variables have also been determined for the fuzzy model (Table 1). Each of the four quadrants of the Cartesian coordinate system was divided into two, resulting in eight sub-quadrant linguistic values (Q1, …, Q8) assigned to the angle variable. The distance variable has three linguistic values: short, medium, and long. The numerator and denominator variables were also assigned three linguistic values, namely, most negative, zero, and most positive.

**Figure 4:**
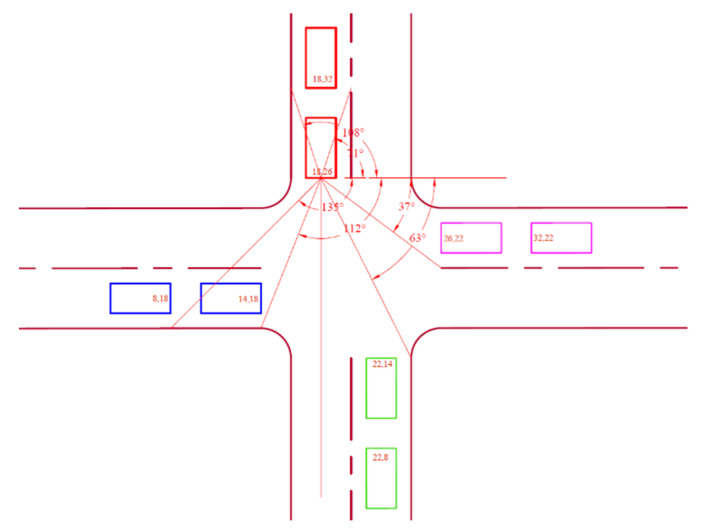
Northern lane angles at Junction A.

Junction A coordinates are shown in Figure 4, displaying angles relative to the leading node N3 on the northern lane.

These angle intervals in Table 2 cover the position a leading vehicle may find itself on each lane as viewed from N3.

**Figure 5:**
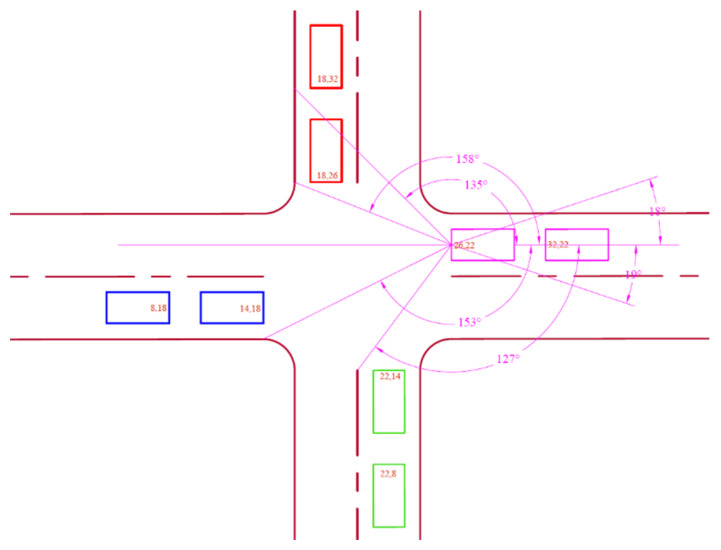
Eastern lane angles at Junction A.

Junction A coordinates are shown in Figure 5, displaying angles relative to the leading node N2 on the eastern lane.

These angle intervals in Table 3 cover the position a leading vehicle may find itself on each lane as viewed from N2.

**Figure 6:**
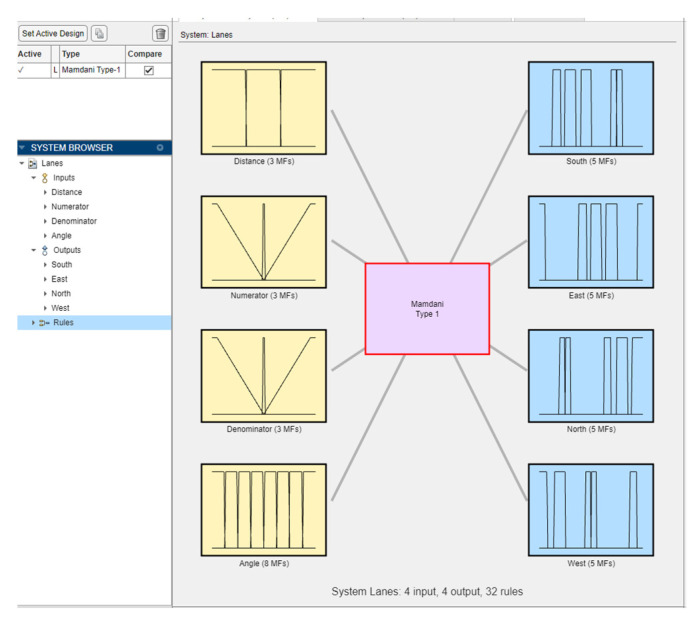
The model in MATLAB Release 2023a fuzzy logic designer.

All mutual characteristics existing between node pairs were computed using MS Excel for the establishment of fuzzy rules. The corresponding fuzzy logic model is shown in Figure 6. The model consists of four input variables, four output variables, and thirty-two rules.

### 4.1. Functional Requirements

All certified road-worthy vehicles possess some inherent capabilities. The autonomous car observes passive control rules including the right-before-left priority (in Germany), like the human driver, and decelerates appropriately upon approaching an intersection. Some of the mandatory requirements in support of system functionality are the following:

1. Accommodation of new vehicles when MAC addresses and registration numbers are provided.
2. Recognition of the relative positions of stopped vehicles at four-legged road network intersections.
3. Coordination of enforcement lights (red, yellow, and green) for single-lane dispersion of autonomous and classic vehicles at four-legged road network intersections.
4. Remaining tolerant to the arrival and departure of vehicles from the GAIM network at intersections.

### 4.2. Non-Functional Requirements

System qualitative attributes also include:

1. A leading vehicle receives priority ahead of a trailing one or concurrently.
2. Single-lane dispersal occurs under thirty seconds of real time.
3. Precision of GPS sensor is kept in centimeters.
4. System reliability, availability, and maintainability should be preserved at all times.

## 5. Implementation and Evaluation

The fuzzy location classification model created with MATLAB 2023a was converted to Arduino C++ and its fis_evaluate() function mapped into the four quadrants of the Cartesian coordinate system. Vehicles on the self-organizing network are identified as nodes which are actually ESP32-WROOM-DA microcontrollers having unique MAC addresses and assigned their hosting vehicle registration numbers with three LEDs attached to them (Table 4).

These microcontrollers have the ability to act as senders and receivers by means of the ESPNow feature which is decoupled from the internet. The station interface of ESP-Now was chosen for sending and receiving data in the system. To effect the data exchange, eight ESP32 board peers were added via their MAC addresses, referred to as broadcast addresses.

Relative vehicle location classification was accomplished in two steps. Step one covered left, right, and ahead positions. Step two covered cars behind and within the Back1 and Back2 locations. The various positions were identified by mapping the fuzzy logic model outputs to corresponding quadrants within the Cartesian coordinates system. The northern lane was selected for vote casting.

A vehicle is deemed to be on the northern lane if:

1. It is on the left of node N4.
2. It is on the right of node N2.
3. It is ahead of node N1.

**Figure 7:**
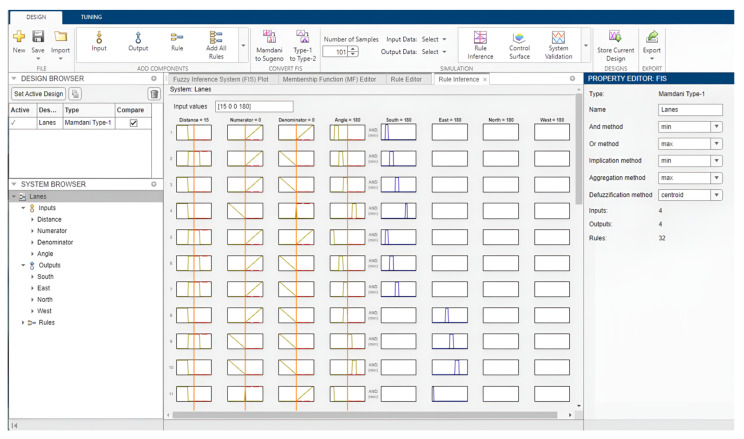
Fuzzy rule viewer in MATLAB Release 2023a.

In MATLAB, the rule viewer (Figure 7) can be used to verify the output of the fuzzy location classification model. Upon adjusting the input values, it allows you to view the effect of each fuzzy rule, the aggregated output fuzzy set, and the defuzzified output value.

As of this writeup, no simulation software exists that supports the ESP-Now data broadcast feature, and testing of the in-vehicle traffic light system is only possible on the physical ESP32-WROOM DA devices.

The accuracy of the GPS click module was tested on the microcontroller at various locations several meters apart and at an unsignalized intersection at Zuschka in Cottbus, Germany. The readings were the same up to three decimal places. Therefore, the GPS click modules could not be relied on to produce a realistic centimeter accuracy needed for the system.

Simulations were carried out using coordinates at Junction A, Junction B, and Junction C (Table 5). Coordinates were shifted to account for Back1 and Back2.

**Figure 8:**
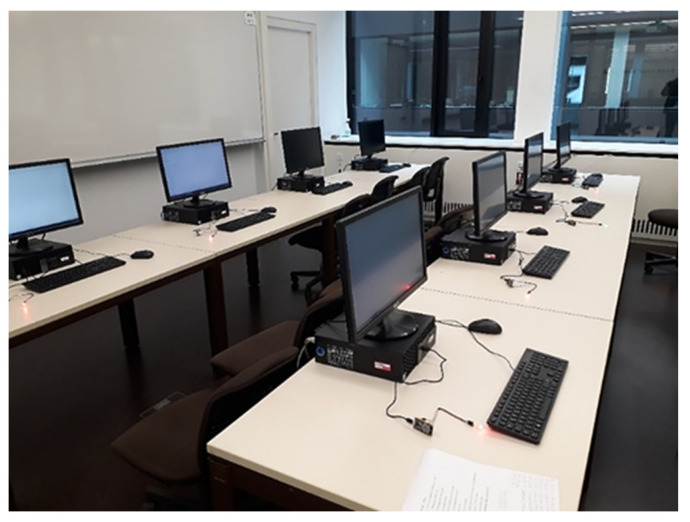
ESP32 microcontrollers with Light-Emitting Diodes.

A car is considered to have arrived at a junction if it turns on one of the three LED lights (Figure 8).

**Figure 9:**
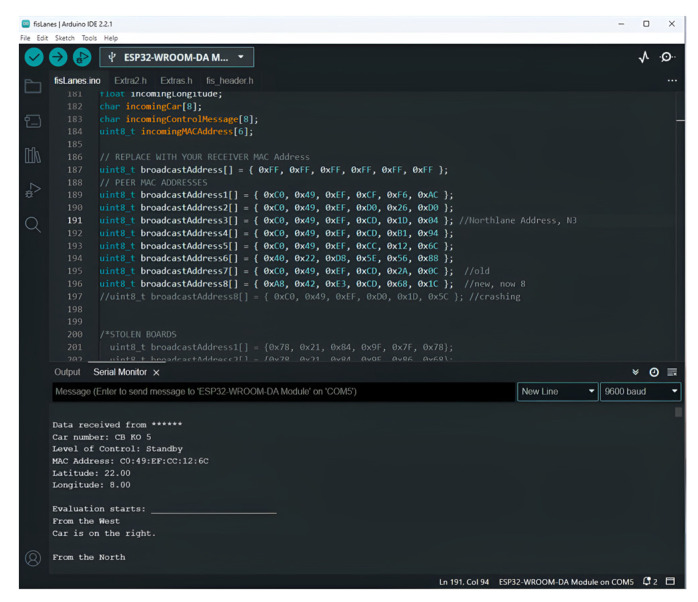
Serial monitor displaying standby and direction of arrival.

**Figure 10:**
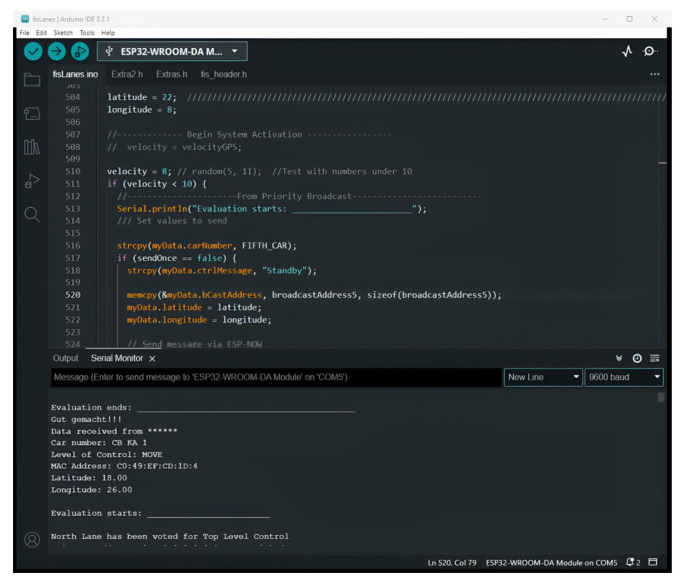
Serial monitor displaying north lane voted to move.

By connecting the microcontrollers to computers, the serial monitor is able to indicate the broadcast coordinates, the level of control, and the direction of arrival (Figure 9), as well as the result of voting for vehicles on the northern lane (leading vehicle) (Figure 10) as the location data are broadcast.

The Road Traffic Gesture Autonomous Integrity Monitoring technology was evaluated based on its ability to:

1. Identify relative vehicle positions at the junctions.
2. Vote for a single lane to disperse.
3. Adapt to changes in the arrival order.
4. Adapt to changes in the arrival direction.
5. Accommodate the removal and addition of nodes to the self-organizing network.

The order of arrival refers to which node (vehicle) arrives at the intersection before the other. The direction of arrival means from the south, east, north, or west. These are determined by the nodes (vehicles) broadcasting different coordinates.

## 6. Results and Discussions

The model gives correct position classification output on the serial monitor in Figure 9. The leading cars (cars in front) were required to vote for the leading car on the northern lane (Figure 10). Each trailing car (car behind) was programmed to interact with the leading car in its lane for effective lane dispersion. Thirty-six arrival patterns (Table 6) out of the five hundred and seventy-six patterns were used in determining the longest dispersion delay in the northern lane. Table 6 displays the range of elapsed times for dispersion in the northern lane.

To establish the longest waiting times during testing, nodes in the northern lane were the last to arrive in both leading and trailing situations (Table 6). Each pattern had the leading northern-lane car as the fourth arrival and the trailing northern-lane car as the eighth arrival. When the thirty-six tests were carried out severally with the coordinates at the three junctions, the leading node always turned green ahead of the trailing node less than fifteen seconds prior to both turning green simultaneously. The two nodes in the northern lane were dispersed during the thirty-six encounters with no single green light popping up from any other lane. This translates into a successful dispersion of nodes in the northern lane. Unlike the conventional traffic light that is based on fixed time allocations, this system is purely self-organizing and priority-based. The earliest dispersion time was 12 s for the leading node and 20 s for the trailing node, averaging the time taken to connect the boards to power sources in simulating the order and direction of arrival. This translates into the successful dispersion of nodes in the northern lane in under 30 s in all cases.

This novel infrastructure-less technology then represents the transfer of the conventional traffic light from the roadside into the car itself based on a self-organizing ad hoc network of vehicles, as the system can be reprogrammed to disperse two opposing lanes simultaneously. The system does not store the location coordinates, and therefore, no expensive, large-scale data storage is needed on-board nor on the driving cloud platform. The low-cost microcontroller is simple to use and easy to implement in classic cars and autonomous vehicles alike. Its hex file is a compiler linked binary code that cannot be easily reversed by hackers. Hence, system security is assured. With this system, runaway vehicles can also be easily identified by the police whenever intersections are crossed. Associated financial benefits to governments cannot be quantified, as it represents the cheapest means of making all four-legged road intersections signalized at zero cost. With the average installation and annual operating costs of traffic lights in the U.S. pegged at USD 33 billion and USD 780 million, respectively [20], huge savings would be accrued by governments on a global scale.

This technology further paves way for an extended blind-spot detection on highways: a solution sought after by automobile manufacturers. Each vehicle can now determine the location of cars ahead, on its left, on its right, or behind simply by sharing its GPS location coordinates with other vehicles, even under bad weather, occlusion, and in poorly lit environments, all without the use of the internet. An extended blind-spot detection technology will caution drivers of cars ahead, especially behind trucks and loaded obstacles, in addition to cars on the left, on the right, and behind at a very low cost. In effect, it will help to prevent head-on collisions arising from sudden lane departures, saving lives and properties.

The novel technology introduces a new algorithm derived from original ideas to relocate roadside traffic devices into autonomous and classic vehicles. As a very promising pioneering strategy for conflict resolution at intersections, this in-vehicle traffic control system stands to open up opportunities for innovative research in academia and industry with huge benefits in uncharted territories using fuzzy logic.

## 7. Conclusions

The Road Traffic Gesture Autonomous Integrity Monitoring in-vehicle traffic control system has been implemented for resolving gridlock conflicts involving autonomous and classical vehicles at four-legged intersections. After delineating the mutual characteristics between pairs of vehicle location coordinates, a fuzzy logic classification model was implemented that has a 100% accuracy in identifying the relative positions of vehicles ahead, on the right side, on the left side, and on the back side of each vehicle. Eight ESP32 WROOM DA microcontrollers embedded with the ESP-Now feature were chosen for forming the intended vehicular network. Based on their assigned registration and MAC addresses, the eight boards running the same program were able to communicate and vote for vehicles in the northern lane to disperse. Two cars in the northern lane were dispersed under thirty-six possible arrival patterns with no extra node turning green from the remaining six nodes running the same program. When the coordinates were reshuffled to simulate the order and direction of arrival, the system performed as expected. The scalable, self-organizing network is able to admit new boards and disconnect them safely without degrading performance.

The scalable system employs a single sensor whose functionality does not degrade under bad weather and requires only the addition of a MAC address and vehicle registration number when admitting new cars. The recommendation for a more effective centimeter-precision GPS input to the system cannot be overemphasized. Perhaps a military-grade precision sensor with such capabilities can be made available to evaluate the performance of the Road Traffic Gesture Autonomous Integrity Monitoring system. While a GPS sensor may receive updates on the health status of satellites in orbit, the sensor itself does not give updates on its health status. It is recommended that the health status of sensors is further researched for the long-term impact on operation of the in-vehicle traffic light system. This will serve as a warning and help in preventive maintenance. Priority is indicated by three LED lights. The serial monitor outputs texts that indicate results of relative vehicle location classification. An on-screen dial indicator on a tablet could be introduced to show the locations of nearby vehicles ahead, on the right, and on the left side to enhance the system output. This will aid in blind-spot detection at intersections, as well. In-vehicle displays can convey more information to the intimate space of the driver than a glance at roadside traffic lights. Integrating the system with existing in-vehicle networks could make diagnostic fault codes easily available for system improvement and maintenance.

Successful simulations point to the fact that the cheap, infrastructure-less in-vehicle traffic control technology would lead to lightweight algorithms for autonomous vehicles to communicate with human drivers and be able to turn all four-legged road intersections into signalized ones at zero cost to governments.

## Figures and Tables

**Table 1:** Fuzzy variable definition.

Linguistic Variables	Linguistic Values								
	*Input*								
Distance	Short			Medium		Long			
Numerator	Most negative			Zero		Most positive			
Denominator	Most negative			Zero		Most positive			
Angle	Q1	Q2	Q3	Q4	Q5	Q6	Q7		Q8
	*Output*								
South	Right		Ahead	Left		Back1		Back2	
East	Back1		Right	Ahead		Left		Back2	
North	Back1		Back2	Right		Ahead		Left	
West	Ahead		Left	Back1		Back2		Right	

**Table 2:** Leading node positions viewed from the northern lane.

	Northern Lane Viewpoints		
Angle Interval (°)	225–248	270–297	323–360
Label	Right	Ahead	Left

**Table 3:** Leading node positions viewed from the eastern lane.

	Northern Lane Viewpoints		
Angle Interval (°)	135–158	180–207	233–270
Label	Right	Ahead	Left

**Table 4:** Node, MAC address, and assigned vehicle registration number.

Car Node	MAC Address	Car Registration Number
N1	C0:49:EF:CF:F6:AC	CB KA 1
N2	C0:49:EF:D0:26:D0	CB AK 2
N3	C0:49:EF:CD:1D:04	CB OA 3
N4	C0:49:EF:CD:B1:94	CB AO 4
N5	C0:49:EF:CC:12:6C	CB KO 5
N6	40:22:D8:5E:56:88	CB OK 6
N7	C0:49:EF:CD:2A:0C	CB OK 7
N8	C0:49:EF:D0:1D:5C	CB OK 8

**Table 5:** Junctions with location coordinates.

Junction B			Junction C		
	65, 65			90, 25	
	69, 73			94, 33	
	61, 77			86, 37	
	57, 69			82, 29	
65.5, 59	65, 59	64.5, 59	89.5, 19	90, 19	90.5, 19
75, 73.5	75, 73	75, 72.5	100, 32.5	100, 33	100, 33.5
61.5, 83	61, 83	60.5, 83	85.5, 43	86, 43	86.5, 43
51, 69.5	51, 69	51, 68.5	76, 28.5	76, 29	76, 29.5

**Table 6:** Evaluation of results.

Order of Arrival											
Patterns	Leading Nodes				Trailing Nodes				Elapsed Time (s)		
									Leader	Trailer	Both
1	1	4	2	3	5	8	6	7	<15	<22	<30
2	1	4	2	3	5	8	6	7	<15	<22	<30
3	1	4	2	3	6	8	5	7	<15	<22	<30
4	1	4	2	3	6	5	8	7	<15	<22	<30
5	1	4	2	3	8	5	6	7	<15	<22	<30
6	1	4	2	3	8	6	5	7	<15	<22	<30
7	1	2	4	3	5	8	6	7	<15	<22	<30
8	1	2	4	3	5	6	8	7	<15	<22	<30
⋮	⋮	⋮	⋮	⋮	⋮	⋮	⋮	⋮	⋮	⋮	⋮
35	4	2	1	3	8	5	6	7	<15	<22	<30
36	4	2	1	3	8	6	5	7	<15	<22	<30

## Data Availability

Data are contained within the article.
